# Improving of Rice Blast Resistances in *Japonica* by Pyramiding Major R Genes

**DOI:** 10.3389/fpls.2016.01918

**Published:** 2017-01-03

**Authors:** Ning Xiao, Yunyu Wu, Cunhong Pan, Ling Yu, Yu Chen, Guangqing Liu, Yuhong Li, Xiaoxiang Zhang, Zhiping Wang, Zhengyuan Dai, Chengzhi Liang, Aihong Li

**Affiliations:** ^1^Lixiahe Agricultural Research Institute of Jiangsu Province, Yangzhou – Jiangsu Collaborative Innovation Center for Modern Crop Production, Nanjing – Institute of Jiangsu Province National Rice Industry Technology System of Yangzhou Comprehensive Experimental StationYangzhou, China; ^2^Institute of Genetics and Developmental Biology, Chinese Academy of SciencesBeijing, China

**Keywords:** *Japonica* rice, blast resistance, polygene pyramid lines

## Abstract

Rice blast, caused by the fungal pathogen *Magnaporthe oryzae*, is a major constraint to rice production worldwide. In this study, we developed monogenic near-isogenic lines (NILs) NIL^*Pi*9^, NIL*^Pizt^*, and NIL^*Pi*54^ carrying genes *Pi9*, *Pizt*, and *Pi54*, respectively, by marker assisted backcross breeding using 07GY31 as the *japonica* genetic background with good agronomic traits. Polygene pyramid lines (PPLs) PPL^*Pi*9+*Pi*54^ combining *Pi9* with *Pi54*, and PPL^*Pizt*+*Pi*54^ combining *Pizt* with *Pi54* were then developed using corresponding NILs with genetic background recovery rates of more than 97%. Compared to 07GY31, the above NILs and PPLs exhibited significantly enhanced resistance frequencies (RFs) for both leaf and panicle blasts. RFs of both PPLs for leaf blast were somewhat higher than those of their own parental NILs, respectively, and PPL*^Pizt^*^+^^*Pi*54^ exhibited higher RF for panicle blast than NIL*^Pizt^* and NIL^*Pi*54^ (*P* < 0.001), hinting an additive effect on the resistance. However, PPL^*Pi*9+*Pi*54^ exhibited lower RF for panicle blast than NIL^*Pi*9^ (*P* < 0.001), failing to realize an additive effect. PPL*^Pizt^*^+^^*Pi*54^ showed higher resistant level for panicle blast and better additive effects on the resistance than PPL^*Pi*9+*Pi*54^. It was suggested that major R genes interacted with each other in a way more complex than additive effect in determining panicle blast resistance levels. Genotyping by sequencing analysis and extreme-phenotype genome-wide association study further confirmed the above results. Moreover, data showed that pyramiding multiple resistance genes did not affect the performance of basic agronomic traits. So the way to enhance levels of leaf and panicle blast resistances for rice breeding in this study is effective and may serve as a reference for breeders.

**Key Message:** Resistant levels of rice blast is resulted from different combinations of major R genes, PPL*^Pizt^*^+^^*Pi*54^ showed higher resistant level and better additive effects on the panicle blast resistance than PPL^*Pi*9+*Pi*54^.

## Introduction

Rice (*Oryza sativa*) is a staple food crop for more than 50% of the world’s population. Rice blast is caused by *Magnaporthe oryzae*, a fungus that infects all parts of rice plant but causes the greatest losses when necks and panicles are infected, it has been leading to severe yield losses worldwide and threatening global food security (Liu et al., 2014). Using host resistance has been proven to be the most effective and economical method to control rice blast (Fukuoka et al., 2009). So far, 102 rice blast R genes have been identified (Su et al., 2015; Vasudevan et al., 2015; Zheng et al., 2016). Among them, 27 genes have been cloned: *Pib, Pb1*, *Pita*, *Pi9*, *Pi2*, *Pizt*, *Pid2*, *Pi33*, *Pii*, *Pi36*, *Pi37*, *Pikm*, *Pit*, *Pi5*, *Pid3*, *Pid3-A4*, *Pi54*, *Pish*, *Pik*, *Pikp*, *Pia*, *PiCO39*, *Pi25*, *Pi1*, *Pi21*, *P50* and *Pi65(t)* (Liu et al., 2014; Su et al., 2015; Zheng et al., 2016). The majority of rice blast R genes are associated with a HR according to the gene-for-gene concept, and race specificity is the key feature of this R gene-mediated disease resistance (Fukuoka et al., 2009). Due to highly frequent variation in the *M. oryzae* population (Bonman, 1992), durable resistance of new rice varieties simply with only a major R gene could be lost quickly, especially when such a variety is grown in large areas (McDonald and Linde, 2002). Therefore, to acquire a durable and broad-spectrum resistant variety, pyramiding multiple R genes into a current rice variety is an important and practicable breeding strategy on controlling blast disease (Hittalmani et al., 2000; Fukuoka et al., 2012). However, with so many available blast R genes, methods to pyramid R genes and actual resistance levels of each R gene are still unknown.

*Pi9*, at the *Piz* locus, producing broad-spectrum blast resistance was cloned from chromosome 6 of *Oryza minuta*, a tetraploid wild species of the *Oryza* genus (Zhou et al., 2006). *Pizt*, a multiple allele of *Pi9*, was isolated from rice cultivar Toride 1 (Mackill and Bonman, 1992). *Pi9* and *Pizt* belong to the NBS-LRR class of R genes (Qu et al., 2006). The NBS-LRR class encodes a receptor-like kinase (Chen et al., 2006). *Pi54* (formerly known as *Pi-kh*) was first identified in the *indica* rice cultivar HR22 (Kiyosawa and Murty, 1969), and was cloned from the *indica* rice cultivar Tetep (Sharma et al., 2005). Another donor variety of *Pi54* is K3 (Xu et al., 2008). *Pi54* belongs to the CC-NBS-LRR class of R genes and expresses a protein that can activate several downstream related pathways against pathogen attack. The above R genes confer broad-spectrum resistance to *indica* rice blast isolates (Ballini et al., 2008; Rai et al., 2011; Khanna et al., 2015). However, under *japonica* genetic background, actual levels of blast resistances of *Pi9*, *Pizt*, and *Pi54* had not been reported. Here, we developed monogenic NILs NIL^*Pi*9^, NIL*^Pizt^*, NIL^*Pi*54^, and PPLs PPL^*Pi*9+*Pi*54^, and PPL^*Pizt*+*Pi*54^, respectively, by MABB using 07GY31 as the *japonica* genetic background. This study will not only report an effective way to enhance levels of leaf and panicle blast resistances in rice plants during rice breeding, but also provide important information that levels of panicle blast resistance are actually resulted from different combinations of major R genes, major R genes would not just produce a simple additive effect, gene reaction would also happen.

## Materials and Methods

### Development of NIL^*Pi*9^, NIL*^Pizt^*, NIL^*Pi*54^, PPL^*Pi*9+*Pi*54^, and PPL^*Pi*9+*Pizt*^

**FIGURE 1:**
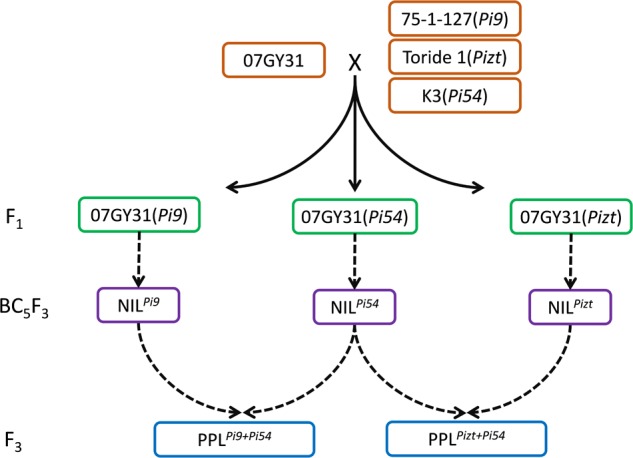
**Development of NIL^*Pi*9^, NIL*^Pizt^*, NIL^*Pi*54^, PPL^*Pi*9+*Pi*54^, and PPL^*Pizt*+*Pi*54^ MAS, marker-assisted selection; NIL, near-isogenic line; PPL, polygene pyramid line**.

Using 75-1-127, Toride 1, and K3 as donor parents of genes *Pi9, Pizt*, and *Pi54*, respectively, and 07GY31, a blast-susceptible *japonica* variety, as the recurrent parent, F_1_ progenies of 75-1-127/07GY31, Toride 1/07GY31, and K3/07GY31 were produced and backcrossed, resulting in three BC_1_F_1_ populations, respectively (**Figure 1**). Markers closely linked with *Pi9*, *Pizt*, and *Pi54*, respectively, were then used to check targeted genes among the above BC_1_F_1_ populations. Twenty plants with the targeted gene from each BC_1_F_1_ population were selected to backcross with the recurrent parent, up to BC_5_F_1_. During the development, no selection for agronomic traits was carried out. After selfing the BC_5_F_1_ and identification of corresponding targeted gene among each BC_5_F_2_ population, 10 plants with homozygous genotype of the targeted gene were selected randomly from each segregating population. BC_5_F_3_ seeds were then harvested individually from each selected BC_5_F_2_ plant, constituting NIL^*Pi*9^, NIL*^Piztt^*, and NIL^*Pi*54^, each featured a blast resistant phenotype.

To further enhance levels of blast resistance, PPLs combining *Pi9* with *Pi54* and PPLs combining *Pizt* with *Pi54* were then developed by MAS (**Figure 1**). Crossing NIL^*Pi*9^ with NIL^*Pi*54^ produced F_1_ with heterozygous genotypes at *Pi9* and *Pi54* loci. After self-pollination, 211 plants from the resulted F_2_ segregating population were used to select lines with both homozygous genotypes at *Pi9* and *Pi54* loci, and nine double homozygous plants were acquired. After determination of genetic background recovery rates, a plant showed the highest genetic background recovery rate (98.52%) was found and named PPL^*Pi*9^^+^^*Pi*54^. Its seeds were then harvested to plant for the evaluation of both leaf and panicle blast resistances. The development process of PPLs combining *Pizt* with *Pi54* was similar to that of PPL^*Pi*9^^+^^*Pi*54^, resulting in a PPL plant, named PPL*^Pizt^*^+^^*Pi*54^, with a genetic background recovery rate of 98.67%. Its seeds were also harvested to plant for the evaluation of both leaf and panicle blast resistances.

To verify whether major-R-gene combination could affect levels of blast resistances, PPL*^Pizt^*^+^^*Pi*54^ and PPL^*Pi*9+*Pi*54^ were selected to cross with 07GY31, respectively. Their F_1_ progenies were subjected to selfing and hence two F_2_ segregating populations were produced, respectively. F_3_ seeds from each F_2_ single plant were individually harvested and used to plant and determine panicle blast resistance levels which would indirectly reflect panicle blast resistance levels of their parent F_2_ plants. All plants were grown in the Yangzhou Wantou experimental fields at the Lixiahe Agricultural Research Institute of Jiangsu Province (119°42′ E, 32°39′ N) and in Sanya City in Hainan Province (110°02′E, 18°48′ N).

### Evaluation of Seedling Leaf and Panicle Blast Resistances for NILs and PPLs

For pathogen collection for the determination of both leaf and panicle blast resistances, *M. oryzae* (rice blast pathogen) isolates were obtained from diseased panicles by single-spore isolation. In total, 112 physiological races of *M. oryzae* were collected, from Anhui, Jiangsu, Hunan, Hubei, Hainan, Henan, and Guangdong, for the leaf blast resistance determination for the above NILs, PPLs, 07GY31, Tetep, Zhenglong 13, Sifeng 43, Dongnong 363, Kanto 51, Hejiang 18, and LTH. Wu et al.’s (2015) method was referred to for leaf blast resistance evaluation. 7 days after inoculation, disease reaction of plant leaves was recorded in accordance with standard methods (Bonman, 1992). Test fields (test nurseries) were located at the experimental fields at the Lixiahe Agricultural Research Institute of Jiangsu Province (119°42′ E, 32°39′N). Resistance levels of lines were measured with RFs. RFs meaning proportions of plants showing resistant phenotype, were calculated according to the following formula: RF = R/(R + S) × 100%, where R is the total number of plants showing resistant phenotype, while S is the total number of plants showing sensitive phenotype. For the determination of panicle blast resistance for the above NILs, PPLs, 07GY31, in total, 64 *M. oryzae* isolates from Anhui, Guangdong, Henan, Hubei, Hainan, Jiangsu, and Zhejiang were utilized. 192 experiment plots were involved in a randomized block design to realize three biological repeats, and 30 plants were grown in each plot. All plants were grown under natural field conditions. One booting panicle was selected for each plant based on the principle that the distance between pulvini of flag leaf and penultimate leaf is 4 cm and was hence injected with 1 mL conidial suspension at a concentration of 5 × 10^4^ conidia/mL (Puri et al., 2009). Panicle blast resistance evaluation was based on the severity of symptoms in infected panicles 30 days after heading according to Titone et al.’s (2015) methods.

All NILs, PPLs, and 07GY31 were also subjected to the determination of blast resistance in blast nurseries in two hot spot fields (one in Xinyi City in Jiangsu Province and the other in Hefei City in Anhui Province). Field rows were involved in a CRBD, and produced three replications. For each line or variety, 60 plants were planted in five rows, meaning 12 plants per row. The evaluation of leaf blast and panicle blast severity in each of the NILs and the recurrent parent was performed using a 0–9 ordinal scale ([IRRI], 2002), where 0–1 = highly resistant, 2–3 = resistant, 4 = mildly resistant, 5–6 = mildly susceptible, 7 = susceptible and 8–9 = highly susceptible, resistant level reflected as RPR according to the following formula: RPR = (HR + R + MR)/60 × 100%, where HR is the total number of plants showing high resistant phenotype, while R is the total number of plants showing resistant phenotype and MR is the total number of plants showing mildly resistant phenotype. RPR were determined 30 days after heading. For further verifying results from the comparison of panicle blast resistance levels of NILs and PPLs, three fields surrounded by virgin land were selected from Jiangsu and Anhui, respectively.

### DNA Extraction and Genotyping

For MAS during each generation, three-week-old rice leaves were individually collected from NIL and PPL plants, and immediately frozen in liquid nitrogen and stored at -80°C for future DNA extraction. Genomic DNA was rapidly extracted by the TPS method for future molecular marker analysis (Rogers and Bendich, 1985). PCR amplification was conducted according to the workflow described by Xiao et al. (2016). Molecular markers closely linked with major Pi genes *Pizt*, *Pi9*, and *Pi54, respectively*, (**Supplementary Table 1**) were used for MAS during the development of each NIL population and each PPL population.

For GBS analysis, genomic DNA was extracted from 100 mg (fresh weight) of three-week-old rice leaf tissue, using the DNA Secure Plant Kit (Qiagen, USA) and following manufacturer’s instruction. Each extracted genomic DNA was qualitatively estimated by electrophoresis on 1% agarose gels, quantitatively measured by Biophotometer Plus (Eppedorf, Germany), and diluted to100 ng/μL with TE buffer. Diluted genomic DNA was then stored at -20°C.

### Genome Alignment and Variant Calling

Genotyping by sequencing was conducted by the Illumina HiSeq^TM^ 2000 system generating 90 bp paired-end read. Raw reads with a mapping quality score of less than 20 were discarded. BWA v0.5.9 (Li and Durbin, 2010) was used to map raw paired-end reads to the *japonica* Os-Nipponbare-Reference-IRGSP-1.0 genome assembly (International Rice Genome Sequencing Project1.0^1^). Alignment files were then input into the GATK V1.2 (McKenna et al., 2010) to identify SNPs. Multiple SNP calling was performed using the GATK Unified Genotyper caller. SNPs with quality scores of >20 and coverage of between two and twice the mean coverage of all accessions were selected. If a SNP was called at the same position in more than one accession, it would be retained. XP-GWAS (Yang et al., 2015)^2^ was then conducted to find out blast-resistance-related QTLs based on the acquired SNP genotypes.

### Evaluation of Agronomic Traits for NILs

To check whether introduction of a major *Pi* gene could affect agronomic traits and select NILs with good agronomic traits, two test sites, one located at the Lixiahe Agricultural Research Institute of Jiangsu Province and the other in Anhui Province, were used to evaluate basic yield-related traits including period from HD to date of 50% flowering, PH, PN, GNP, SR, 1000-GW and YPP, of NILs. The evaluation was performed based on a CRBD with two replications. Each line involving 120 plants was planted in 10 rows. Planting methods were the same as the above. Agronomic traits were measured according to the Standard Evaluation System for Rice (International Rice Research Institute [IRRI], 2002). Five single plants between the second and the sixth rows in each plot were taken for measurements of agronomic traits.

## Result

### NIL*^Pizt^*, NIL^*Pi*54^, and NIL^*Pi*9^ Exhibited Significantly Higher RFs for Both Leaf and Panicle Blasts than 07GY31

**FIGURE 2:**
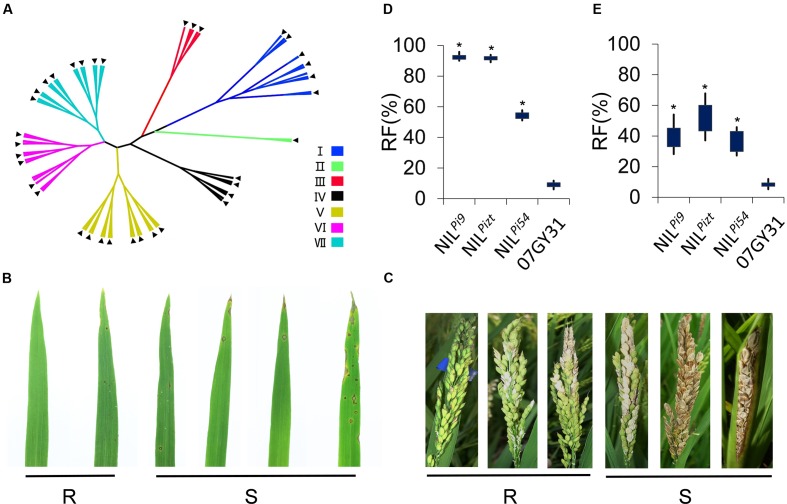
**Evaluation of leaf and panicle blast resistances for NILs and PPLs.** **(A)** Seven groups classification of 112 physiological races based on the resistance responses of varieties Tetep, Zhenlong13, Sifeng4, Dongnong363, Kanto 51, Hejiang18, and LTH; **(B)** Classification of leaf blast resistance; **(C)** Classification of panicle blast resistance; **(D)** Resistance levels of NIL*^Pizt^*, NIL^*Pi*54^, NIL^*Pi*9^, and 07GY31 for leaf blast; **(E)** Resistance levels of NIL*^Pizt^*, NIL^*Pi*54^, NIL^*Pi*9^, and 07GY31for panicle blast. **R**: Resistant; **S**: Susceptible. ^∗^Statistically significant at *P* < 0.001 level; 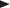:64 selected physiological races for the determination of panicle blast resistance.

Genetic background recovery rates of NIL^*Pi*9^, NIL^*Pi*54^, and NIL*^Pizt^* were found to be 98.25, 97.33, and 97.82%, respectively (**Supplementary Table 2**). These NILs were then subjected to both inoculations of leaf and panicle blasts for the determination of resistances. Rice blast pathogens were collected from 7 regions of China, namely, Jiangsu, Hunan, Anhui, Hubei, Sanya, Henan, and Guangdong. Totally 112 physiological races were involved. Based on resistance responses of varieties Tetep, Zhenlong13, Sifeng4, Dongnong363, Guandong51, Hejiang18, and LTH to the races, we divided these races into seven types, namely, typed I, II, III, IV, V, VI, and VII (**Figure 2A**). Plants rated 0–3 in the identification of resistance to either leaf or panicle blast were considered to be resistant to the corresponding blast and each got an “R,” while plants rated 4–5 each got an “S” (**Figures 2B,C**). All the 112 races were involved in the identification of resistance to leaf blast. Finally, we found RFs of NIL*^Pizt^*, NIL^*Pi*54^, and NIL^*Pi*9^ were significantly enhanced for leaf blast (*P* < 0.001), compared to that of 07GY31. NIL*^Pizt^* and NIL^*Pi*9^ exhibited significantly higher RFs than NIL^*Pi*54^ (*P* < 0.001) (**Figure 2D**). Data indicated that resistance levels of NIL*^Pizt^*, NIL^*Pi*54^, and NIL^*Pi*9^ for panicle blast were also significantly enhanced, compared to that of 07GY31, based on 64 selected to identify resistance responses. Moreover, NIL*^Pizt^* and NIL^*Pi*9^ exhibited significantly higher RF than NIL^*Pi*54^ (**Figures 2D,E**).

### PPL*^Pizt^*^+^^*Pi*54^Exhibited Higher RF for Panicle Blast than PPL^*Pi*9^^+^^*Pi*54^

**FIGURE 3:**
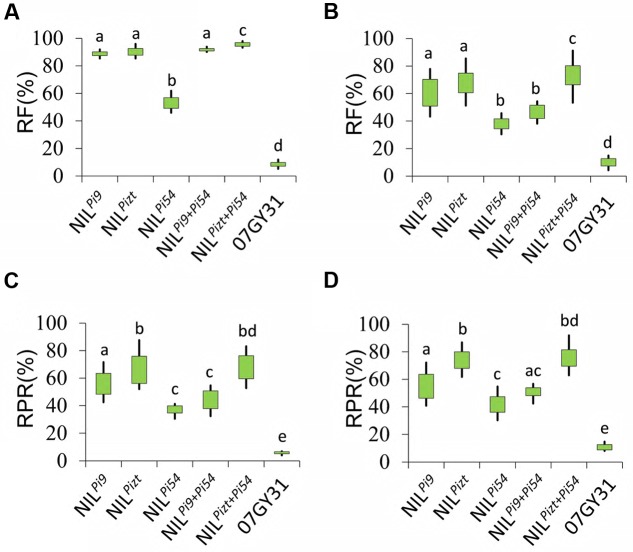
**Blast resistance comparison between NILs and PPLs.** **(A)** Resistance frequencies of NILs, PPLs, and 07GY31 for leaf blast; **(B)** Resistance frequencies of NILs, PPLs, and 07GY31 for panicle blast; **(C)** Resistance levels of NIL*^Pizt^*, NIL^*Pi*54^, NIL^*Pi*9^, and 07GY31for panicle blast; **(C)** Resistance levels of NILs, PPLs, and 07GY31 for the disease spectrum at Anhui test site; **(D)** Resistance levels of NILs, PPLs, and 07GY31 for the disease spectrum at Jiangsu test site. Entries with different letters were statistically significantly different at *P* < 0.001 level; RPR; RF.

Pyramiding multiple major resistance genes is one of the main methods to enhance variety resistance. NIL*^Pizt^* and NIL^*Pi*9^ were then crossed with NIL^*Pi*54^, respectively. Finally, two polygene pyramid lines, PPL*^Pizt^*^+^^*Pi*54^ and PPL^*Pi*9+*Pi*54^, were developed using MAS. Data showed that RFs of all the PPLs for leaf blast were somewhat higher than those of their own parental NILs, respectively, (**Figures 3A,B**), and PPL*^Pizt^*^+^^*Pi*54^ exhibited higher RF for panicle blast than NIL*^Pizt^* and NIL^*Pi*54^ (*P* < 0.001), hinting an additive effect on the resistance. However, PPL^*Pi*9+*Pi*54^ exhibited lower RF for panicle blast than NIL^*Pi*9^ (*P* < 0.001), failing to realize an additive effect. For further verification, two fields surrounded by virgin land were selected for the determination of resistance to panicle blast from Anhui and Jiangsu, respectively. Interestingly, PPL*^Pizt^*^+^^*Pi*54^ showed higher resistant level for panicle blast and better additive effects on the resistance than PPL^*Pi*9+*Pi*54^ (**Figures 3C,D**). In these fields, PPL^*Pi*9^^+^^*Pi*54^ still showed significantly lower resistant level for panicle blast than NIL^*Pi*9^ (*P* < 0.001), meaning major R genes interacted with each other in a way more complex than additive effect in determining panicle blast resistance levels.

### Levels of Panicle Blast Resistance Were Determined by Major Gene Combinations

**FIGURE 4:**
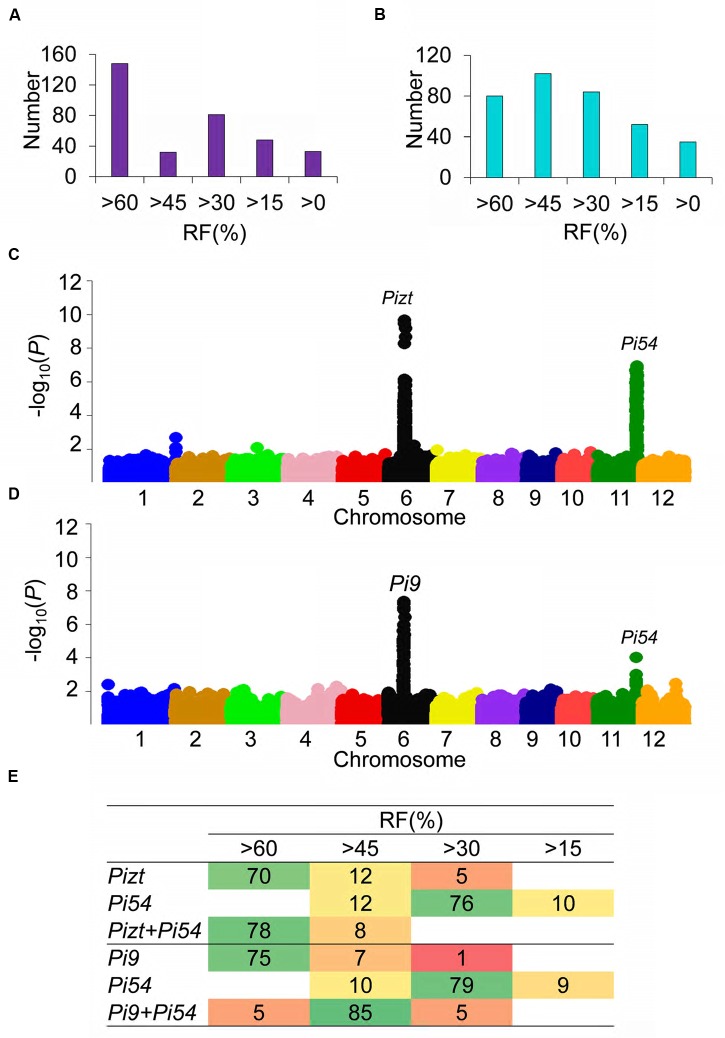
**Extreme-phenotype genome -wide association study of QTL loci related to panicle blast resistance.** **(A)** Panicle blast resistance classification in the F_2_ segregating population of PPL*^Pizt^*^+^^*Pi*54^/07GY31; **(B)** Panicle blast resistance classification in the F_2_ segregating population of PPL^*Pi*9^^+^^*Pi*54^/07GY31; **(C)** XP-GWAS of panicle-blast-resistance-related loci in the F_2_ segregating population of PPL*^Pizt^*^+^^*Pi*54^/07GY31; **(D)** XP-GWAS of panicle-blast-resistance-related loci in the F_2_ segregating population of PPL^*Pi*9^^+^^*Pi*54^/07GY31. **(E)** Distributions of *Pi9*, *Pizt*, and *Pi54* in the F_2_ segregating population of PPL^*Pizt*+*Pi*54^/07GY31 and the F_2_ segregating population of PPL^*Pi*9+*Pi*54^/07GY31. Numbers in the table of **Figure 4E** is the number of plants with *Pi9*, *Pizt*, *Pi54*, *Pizt+Pi54*, *Pi9+Pi54*.

For further verifying the above results, three physiological races were selected from each of the following 7 regions: Jiangsu, Hunan, Anhui, Hubei, Henan, Guangdong, and Sanya. These 21 physiological races were involved in determining the resistance level of each F_2_ single-plant in each F_2_ segregating population (**Figure 4A**). Panicle blast resistance of each F_2_ single-plant was reflected by RFs of its 20 offspring F_3_ plants. F_2_ segregating population of PPL^*Pi*9^^+^^*Pi*54^/07GY31 was constituted by 342 single plants, and F_2_ segregating population of PPL*^Pizt^*^+^^*Pi*54^/07GY31 was constituted by 353 single plants. 120 single F_2_ plants with RFs of either more than 60% or less than 15% were selected from each F_2_ segregating population to construct resistant and susceptible pools; each pool contained 60 single plants. Each mixed pool was then subjected to GBS. Raw reads of each pool were individually assembled using the Os-Nipponbare-Reference-IRGSP-1.0 genome assembly as the reference genome. A sequence with a total length of 3.3 Gb, an average sequencing depth of 5.8× and a coverage of 91.2% over the reference genome, and totally 49,366 SNPs were used to perform QTL mapping for each F_2_ segregating population.

As shown in **Figure 4B**, two strong peaks were detected for the F_2_ segregating population of PPL*^Pizt^*^+^^*Pi*54^/07GY31, indicating that only two major genes would affect the resistance to panicle blast in this population. One peak was found to be associated with the range from 10.1 to 11.29 Mb on chromosome 6, and this range completely covered gene *Pizt* (*P* = 2.26 × 10^-10^); and the other peak was found to be associated with the range from 24.98 to 25.96 Mb on chromosome 11, this range completely covered gene *Pi54* (*P* = 1.19 × 10^-9^) (**Figure 4C**). The above indicated that *Pizt* and *Pi54* should be the only two genes determining panicle blast resistance in the F_2_ segregating population of PPL*^Pizt^*^+^^*Pi*54^/07GY31.

We further used tightly linked markers to scan the genome of each plant and work out the distributions of *Pizt* and *Pi54* in the F_2_ segregating population of PPL*^Pizt^*^+^^*Pi*54^/07GY31. As shown in **Figure 4E**, RFs of more than 60% mainly involved plants with mono-gene *Pizt*, and plants pyramiding both *Pizt* and *Pi54*, while RFs varying from 30 to 45% mainly involved plants with mono-gene *Pi54*. These distributions indicated that pyramiding both *Pizt* and *Pi54* significantly enhanced panicle blast resistance levels.

Interestingly, in the F_2_ segregating population of PPL^*Pi*9+*Pi*54^/07GY31, only one strong peak (*P* = 4.46 × 10^-8^) was detected (**Figure 4D**). This peak was found to be associated with the range from 10.09 to 10.47 Mb on chromosome 6, and this range completely covered gene *Pi9*. Besides this strong peak, only one weak peak (*P* = 8.12 × 10^-3^) was detected, this peak was associated with the range from 24.87 to 25.99 Mb on chromosome 11, and this range completely covered gene *Pi54*.

Tightly linked markers were also used to scan the genome of each plant and work out the distributions of *Pi9* and *Pi54* in the F_2_ segregating population of PPL^*Pi*9^^+^^*Pi*54^/07GY31. Different from the F_2_ segregating population of PPL*^Pizt^*^+^^*Pi*54^/07GY31, RFs of more than 60% in the F_2_ segregating population of PPL^*Pi*9^^+^^*Pi*54^/07GY31 mainly involved plants with mono-gene *Pi9*, while RFs ranging from 40 to 60% mainly involved plants pyramiding both *Pi9* and *Pi54* (**Figure 4E**). PPL*^Pizt^*^+^^*Pi*54^ showed higher resistant level for panicle blast and better additive effects on the resistance than PPL^*Pi*9+*Pi*54^. Therefore, we confirmed that rice plant resistance to panicle blast was directly determined by the combination of resistance genes.

### Introduction of Resistance Gene(s) Had No Effects on Basic Agronomic Traits

NILs, PPLs, and 07GY31 were subjected to the phenotyping of the following basic agronomic traits: growth period, 1000-grain weight, effective panicles per plant, grain number per panicle, seed setting rate, and yield. Phenotypic data did not indicate any significant difference between other lines and 07GY31, except the chalkiness of NIL*^Piz^* and PPL^*Pizt*+*Pi*54^ was higher than 07GY31. It was suggested that significant enhancement of rice blast resistance levels did not affect levels of else agronomic traits, since genetic background recovery rates of the above NILs were high (**Table 1**).

**Table 1:** Performance of basic agronomic traits in NILs and PPLs.

	Plant height(cm)	Heading days(d)	Panicle number	Grain number per panicle	Seeding rate (%)	1,000 grain weight (g)	Yield (kg)	Head rice rate	Chalkiness rate	Amylose content
NIL*^Pizt^*	100.45 ± 3.04a	98 ± 1.41a	12.15 ± 0.64a	129.25 ± 6.15a	91.85 ± 0.78a	25.15 ± 0.36a	551.2 ± 7.07a	74.15 ± 0.78a	31.85 ± 0.78b	16.9 ± 0.85a
NIL^*Pi*54^	99.2 ± 1.84a	97.5 ± 0.71a	12.25 ± 1.34a	132.85 ± 7.42a	92.85 ± 0.92a	25.45 ± 0.35a	561.5 ± 12.02a	73.15 ± 0.92a	22.65 ± 4.17a	17.45 ± 1.49a
NIL^*Pi*9^	100.35 ± 1.49a	96.5 ± 0.701a	12.65 ± 0.21a	124.70 ± 3.25a	91.95 ± 1.06a	25.2 ± 0.85a	561.5 ± 7.78a	74.35 ± 0.21a	24.45 ± 0.92a	16.35 ± 0.78a
PPL^*Pizt*+*Pi*54^	99.5 ± 3.39a	97.5 ± 0.71a	11.95 ± 0.35a	130.75 ± 7.71a	91.7 ± 1.27a	25.45 ± 0.49a	562 ± 19.79a	74.15 ± 0.49a	35.45 ± 4.17b	16.75 ± 0.78a
PPL^*Pi*9+*Pi*54^	101.35 ± 1.48a	98.2 ± 0.6a	12.65 ± 1.49a	125.65 ± 4.31a	92.4 ± 1.13a	25.1 ± 0.57a	553 ± 4.24a	74.35 ± 0.35a	26.2 ± 1.27a	16.15 ± 0.79a
07GY31(CK)	99.45 ± 0.78a	98.2 ± 0.61a	11.75 ± 0.49a	128.65 ± 2.62a	92.15 ± 0.35a	25.05 ± 0.35a	556 ± 8.49a	73.55 ± 0.92a	24.75 ± 4.45a	16.1 ± 1.42a


*Entries with different letters were statistically significantly different at *P* < 0.001 level.*

## Discussion

Rice blast is one of the most destructive diseases. Based on related rice developmental stages, this disease is divided into leaf blast and panicle blast. Panicle blast is highly concentrated on by breeders and geneticists, because it is directly related to paddy rice production safety and rice quality. Pyramiding rice blast resistance genes has been becoming an effective strategy to develop a new variety with long lasting resistance. Currently, several broad-spectrum resistance genes such as *Pi9*, *Pi2*, *Pizt*, and *Pi54*, have already been cloned, but their distribution frequencies in elite parents of Chinese *indica* and *japonica* rice were low (Huang et al., 2015; Wu et al., 2015; Tian et al., 2016). Moreover, research in levels of resistances to leaf and panicle blasts of rice plants pyramiding either *Pi9* or *Pizt*, and *Pi54* hasn’t yet been reported. This study introduced *Pi9*, *Pi54*, and *Pizt* into 07GY31, respectively. Resulted monogenic NILs showed significantly enhanced levels of resistances to leaf and panicle blasts. Further, gene pyramiding was conducted in order to realize the enhancement of resistance levels. Resulted lines pyramiding either *Pizt* or *Pi9*, and *Pi54* exhibited an additive effect on the resistance to leaf blast, compared to corresponding NILs. However, resistance levels of panicle blast were overall lower than those of leaf blast, similar to result reported by Wu et al. (2016). It might be because the pathogenetic process of panicle blast involved the development process of spike types and the rice maturation process, and ultimately, levels of resistance to panicle blast were comprehensive responses of plants to panicle-neck blast, grain blast and spike-neck blast, while leaf blast was simpler. Therefore, overall symptoms of panicle blast were prone to be more serious. Moreover, seedling leaf and young panicle are two different organs at different developmental stages; organ-specific genes might also affect plant resistances. For example, *miR156* was specifically highly expressed in young panicles of rice, and existed along with the whole development process of young panicles (Wang et al., 2010). Coincidentally, in wheat, expression levels of *miR156* had been reported to be related to levels of resistance to wheat powdery mildew caused by a fungal pathogen (Xin et al., 2010, and Bej and Basak, 2014). In additional, *OsRac1*, *WRKY19*, *OsGF14b*, and *MAPK3*/*6* have been identified as downstream genes of R genes participate in resistance regulation (Liu et al., 2014). Liu et al. (2016) also reported low correlation between the levels of leaf and panicle blast resistance observed in the field, and provided that *WRKY71* up-regulates the expression of *OsGF14b* by combining with the promoter of *OsGF14b*, which resulted in the level of panicle blast resistance is enhanced while the level of seedling leaf blast resistance is lowered. Therefore, it’s suggesting that organ-specifically expressed genes and downstream-regulating genes play an important role on blast resistant difference between seedling and panicle.

In this study, we also observed that lines pyramiding both *Pizt* and *Pi54* showed an additive effect on resistance to panicle blast, while lines pyramiding both *Pi9* and *Pi54* exhibited lower resistance levels than *Pi9* monogenic lines. Similar phenomena had been reported by Wang et al. (1994), negative interaction wherein some combinations of R genes actually cause low resistance, lines pyramiding both *Piz5* and *Pizt* showed lower resistance levels than *Piz5* monogenic lines in natural disease nurseries in IRRI (Los Banos, Laguna, Philippines) and India (Hittalmani et al., 2000). Therefore, pyramiding resistance genes did not always lead to enhanced resistance levels (Tabien et al., 2000), different combinations of resistance genes might directly resulted in various resistance levels. It is a pity that no follow-up validation studies have been reported. In this study, we had already located panicle blast resistance QTLs of the F_2_ segregating population of PPL*^Pizt^*^+^^*Pi*54^/07GY31 and the F_2_ segregating population of PPL^*Pi*9+*Pi*54^/07GY31. Data confirmed that PPL*^Pizt^*^+^^*Pi*54^ were more resistant to panicle blast than PPLs^*Pi*9^^+^^*Pi*54^. Therefore, it’s suggest that differences in molecular immune mechanisms existing between *Pi9* and *Pizt*. Rice blast major resistance genes express proteins belong to the NOD-like receptor NLR family (nucleotide-binding domain leucine-rich repeat containing). NLRs can activate downstream gene expression programs after recognizing pathogens and hence initiate plant immune responses (Orbach et al., 2000; Qu et al., 2006; Hayashi and Yoshida, 2009). Therefore, genes that are downstream of and interact with major rice blast resistance genes also can affect plant resistance levels. Kawano et al.’s (2010) research mentioned a domain that is in *Pi9* and called NB-ARC. They pointed out that OsRac1 initiated immune reactions after interacting with the NB-ARC domain of *Pi9*, but failed to combine with *Pizt*. Therefore, though *Pi9* and *Pizt* exist as multiple alleles at Piz locus, their resistance mechanisms may have already been differentiated. Wu et al. (2016) reported NILs each combining the genetic background of *indica* rice variety Yangdao6 with one allele (*Pizt*, *Pi2*, *Pigm*, *Pi40*, *Pi9*, or *Piz*) at *Piz* locus, exhibited significantly different levels of panicle blast resistance. This result is similar to our result that resistance levels of NIL^*Pi*9^ and PPL^*Pi*9+*Pi*54^ were more significantly different than those of NIL*^Pizt^* and PPL*^Pizt^*^+^^*Pi*54^. Therefore, different major resistance genes may determine different molecular immune pathways, and which major resistance genes to select and pyramid becomes the key question during designing molecular breeding.

During MABB period, backcrossing is one of the most common practices for removing donor parent chromosomes both linked and unlinked to the target gene. However, large linkage drags always found in high backcross generation. A fragment with 6.4 Mb around the blast R gene *Pi33* from a wild rice was found in IR64 introgression lines (Ballini et al., 2007). And 11.6 Mb of chromosomal fragment around *Pita* from donor (Tetep) was also identified in BC_5_F_2_ individuals (Jia, 2009). Recently, some study also reported that most agriculture traits of NILs with high background recover rate were similar with recurrent parents, but some relating to grain quality traits (gel consistency, amylose content, etc.), heading date and plant height could be altered (Jiang et al., 2015; Khanna et al., 2015), it’s suggest that some genes or QTLs from the linkage drags influence on traits under receptor genetic background. Similar observation also present in our study, all NILs and PPLs that the genetic background recovery rates of are over 97% failed to exhibit significantly lower levels of yield trait than 07GY31, except the chalkiness rate of NIL*^Pizt^* and PPL*^Pizt^*^+^^*Pi*54^ is higher than 07GY31. Since Toride 1, the donor of *Pizt* gene, possess higher chalkiness rate than 07GY31, some fragments impressing chalkiness as linkage drags introduced into 07GY31 during the MABB process. Anyway, the goal of MABB is recover phenotypically similar if not better improved lines than that of the receptor parent, with all desired plant type and grain quality, in short span of selection time. In our study, PPL*^Pizt^*^+^^*Pi*54^ exhibited higher levels of resistances to leaf and panicle blasts than PPL^*Pi*9+*Pi*54^. These results are already enough to provide a theoretical basis for deciding which major resistance genes to select and pyramid for developing a highly resistant variety. PPL*^Pizt^*^+^^*Pi*54^ showed the highest levels of resistances and the highest yield in the multiple-environment trial. Potential material for future blast resistance breeding is already available.

## Author Contributions

NX, YW, AL, and CL participated in the study conception and design. LY, CP, and YC contributed to DNA extraction and molecular marker identification. GL, YL, XZ, ZW, and ZD contributed to data analysis. NX wrote the manuscript. AL and LZ critically revised the manuscript. All authors approved the final version of the manuscript.

## Conflict of Interest Statement

The authors declare that the research was conducted in the absence of any commercial or financial relationships that could be construed as a potential conflict of interest.

## Footnotes

1 http://rapdb.dna.affrc.go.jp/download/irgsp1.html

2 https://github.com/schnablelab/XP-GWAS

## Abbreviations

BWA: Burrows-Wheeler Aligner
CC-NBS-LRR: coiled coil-nucleotide binding site-leucine rich repeats
CRBD: completely randomized block design
GATK: Genome Analysis Toolkit
GBS: genotyping by sequencing
GNP: grain number per plant
GW: 1000-grain weight
GWAS: genome-wide association study
HD: heading day
HR: hypersensitive response
IRRI: International Rice Research Institute
LTH: Lijiangxintuanheigu
MABB: marker assisted backcross breeding
MAS: marker associated selection
NB-ARC: nucleotide-binding adaptor shared by APAF-1, R proteins, and CED-4
NBS-LRR: nucleotide binding site-leucine rich repeat
NILs: near-isogenic lines
NLR: NOD-like receptor
NOD: nucleotide-binding oligomerization domain
PH: plant height
PN: panicle number per plant
PPLs: polygene pyramid lines
RFs: resistance frequencies
RPR: resistant panicle rate
SNPs: single nucleotide polymorphisms
SR: seeding rate
XP-GWAS: extreme-phenotype genome-wide association study
YPP: yield per plant
