# Supplementary material for: Improving of Rice Blast Resistances in Japonica by Pyramiding Major R Genes
Source: Front Plant Sci. 2017 Jan 3;7:1918. doi: 10.3389/fpls.2016.01918 (PMC5206849; doi:10.3389/fpls.2016.01918)
Supplement: Supplementary file 1 [file Table_1.pdf]

Supplementary Table 1 Primer sequences of molecular markers closely linked with *Pi9*, *Pizt* and *Pi54*.

Gene	Marker Name	Primer sequence (5′–3′)	Annealing temperature (℃)
Pi9	Z4794	F: TGAATGTGAGAGGTTGACTGTGG	55
		R: CACGCCACCCTTCAATGGAGACT	
Pizt	AP22	F: GTGCATGAGTCCAGCTCAAA	58
		R: GTGTACTCCCATGGCTGCTC	
Pi54	pi-k^h	F: CAATCTCCAAAGTTTTCAGG	55
		R: GCTTCAATCACTGCTAGACC	
