# Supplementary material for: Improving of Rice Blast Resistances in Japonica by Pyramiding Major R Genes
Source: Front Plant Sci. 2017 Jan 3;7:1918. doi: 10.3389/fpls.2016.01918 (PMC5206849; doi:10.3389/fpls.2016.01918)
Supplement: Supplementary file 2 [file Table_2.pdf]

Supplementary Table 2 The chromosome elements from donor genomic in 07GY31

	Chr01	Chr02	Chr03	Chr04	Chr05	Chr06	Chr07	Chr08	Chr09	Chr10	Chr11	Chr12	Genetic background recover rate (%)
NIL^{Pizt}	23.21–25.22	/	4.54–5.43	15.21–17.43	/	9.82–12.01	/	/	/	/	/	3.43–4.25	97.82
NIL^{Pi54}	9.52–12.34	7.23–9.32	/	/	/	/	/	22.32–25.21	/	/	23.87–26.01	/	97.33
NIL^{Pi9}	34.12–36.17	18.32–20.43	/	/	/	10.03–12.39	/	/	/	/	/	/	98.25
PPL$^{Pizt+Pi54}$	23.12–24.23	/	4.76-5.32	16.78-17.12	/	10.11–11.29	/	/	/	/	24.98–25.96	3.45–4.22	98.67
PPL$^{Pi9+Pi54}$	11.50–12.32	7.25–8.12	/	/	/	10.11–10.51	/	22.72–25.11	/	/	24.87–25.91	/	98.52

Size: Mb; /: No elements
